# Evaluations of Dentists on a German Physician Rating Website: An Analysis of the Ratings

**DOI:** 10.2196/jmir.3830

**Published:** 2015-01-12

**Authors:** Martin Emmert, Frank Halling, Florian Meier

**Affiliations:** ^1^Institute of Management (IFM)School of Business and EconomicsFriedrich-Alexander-University Erlangen-NurembergNurembergGermany; ^2^Maxillofacial surgeon and dentist, Gesundheitszentrum FuldaGerloser Weg 23a, 36039 FuldaDepartment of Oral and Maxillofacial Surgery, University of Marburg, Baldingerstraße, 35037 MarburgFuldaGermany

**Keywords:** physician rating website, dentist, patient experience, Internet, quality of care

## Abstract

**Background:**

Physician rating websites have been gaining in importance in both practice and research. However, no evidence is available concerning patients’ ratings of dentists on physician rating websites.

**Objective:**

The aim of this study is to present a comprehensive analysis of the ratings of dentists on a German physician rating website over a 2-year period.

**Methods:**

All dentist ratings on a German physician rating website (Jameda) from 2012 and 2013 were analyzed. The available dataset contained 76,456 ratings of 23,902 dentists from 72,758 patients. Additional information included the overall score and subscores for 5 mandatory questions, the medical specialty and gender of the dentists, and the age, gender, and health insurance status of the patients. Statistical analysis was conducted using the median test and the Kendall tau-b test.

**Results:**

During the study period, 44.57% (23,902/53,626) of all dentists in Germany were evaluated on the physician rating website, Jameda. The number of ratings rose from 28,843 in 2012 to 47,613 in 2013, representing an increase of 65.08%. In detail, 45.37% (10,845/23,902) of dentists were rated once, 43.41% (10,376/23,902) between 2 and 5 times, and 11.21% (2681/23,902) more than 6 times (mean 3.16, SD 5.57). Approximately 90% (21,324/23,902, 89.21%) of dentists received a very good or good overall rating, whereas only 3.02% (721/23,902) were rated with the lowest scores. Better ratings were given either by female or older patients, or by those covered by private health insurance. The best-rated specialty was pediatric dentistry; the lowest ratings were given to orthodontists. Finally, dentists were rated slightly lower in 2013 compared to 2012 (*P*=.01).

**Conclusions:**

The rise in the number of ratings for dentists demonstrates the increasing popularity of physician rating websites and the need for information about health care providers. Future research should assess whether social media, especially Web-based ratings, are suitable in practice for patients and other stakeholders in health care (eg, insurance providers) to reflect the clinical quality of care.

## Introduction

Over the past 20 years, the Internet has increasingly gained in importance and has acquired a key role in society as a central means of communications and an information platform. At the beginning of 2014, 76.6% of the German population aged 10 years or older used the Internet. Among the group of users aged 10-39 years, this number amounted to nearly 100% [1]. Health issues seem to be a major field of interest. A German study has shown that 37.7% of the population and 64.5% of Internet users use online research to look for health-related information [2]. In the United States, 88% of adults looked for information concerning health issues on the Internet [3]. In another representative survey in Germany, 11% of the people questioned stated that they had researched dental issues on the Internet at least once [4]. In summary, this illustrates a change in the patient’s role from passive receiver to active user of health-related information on the Internet [5].

This may also reflect the trend in patients wishing to be informed more effectively about the content and quality of medical care provision. However, a balanced and robust judgment of the quality of medical treatment is rarely possible for patients because of a lack of publicly accessible information [6]. Family, friends, and acquaintances still appear to be the most important source of information when looking for a doctor [7,8]. Public reporting has helped to increase the transparency of the quality of medical care [9-12], yet this offer is only accepted hesitantly at the moment [13]. A lack of understanding of the offered medical information, a lack of confidence in the information, and several other reasons might explain a patient’s inability to use the existing platforms on the Internet [14].

Although the existing research has increasingly dealt with patient satisfaction, an overall supply of information about doctors, practices, or hospitals is still missing [15]. Because social media are easily available, easy to use, and are used by the majority of the population, a growing number of people share health care experiences online or rate their health care provider on physician rating websites [16]. Of the nearly 28 million German users of physician rating websites, 56% are female and 44% are male [17]. Despite some studies dealing with physician rating websites [3,18-21], neither national nor international research regarding the rating of dentists on physician rating websites have been published. The intention of this paper is to present the first study on dentist ratings on a German physician rating website (Jameda) analyzing (1) the characteristic features of both dentists and patients, (2) the number and distribution of rating results, (3) the influence of characteristics of dentists rated and rating patients on the submitted grades, and (4) the development of dentist ratings on a physician rating website over 2 consecutive years.

## Methods

### Overview

This study extends the results of a previous study in which ratings for all German physicians from the German outpatient sector (except dentists) were evaluated [18]. Physician rating website Jameda was chosen as the data source because it likely plays the most important role in the German physician rating website movement (see [18]).

The total number of ratings in 2012 and 2013 was 76,456 ratings, which were given by 72,758 patients for 23,902 dentists. The dataset contained information concerning gender, age, and medical specification of the dentists, as well as gender, age, and the type of health insurance of the patients. The rating system on Jameda consists of 5 questions, each rated according to the grading system in German schools from 1=very good to 6=insufficient. The questions are about (1) satisfaction with the treatment offered (Q1), (2) information and presentation of facts with regard to illness and treatment (Q2), (3) the relationship of trust with the dentist (Q3), (4) the amount of time spent on a patient’s concerns (Q4), and (5) the friendliness of the dentist (Q5). An average score is derived subsequently on the basis of the 5 single grades.

### Statistical Analysis

All statistical analyses of the data were carried out with SPSS v21.0 (IBM Corp, Armonk, NY, USA). The median test was applied to nonparametric data of groups with different distributions. The Kendall tau-b test was used to analyze the correlation between both the performance of a dentist and the number of ratings per dentist and the number of ratings per patient compared to the mean overall performance given by this patient. Differences were considered to be significant if *P*<.05 and highly significant if *P*<.001.

## Results

### Number and Distribution of Ratings

**Figure 1:**
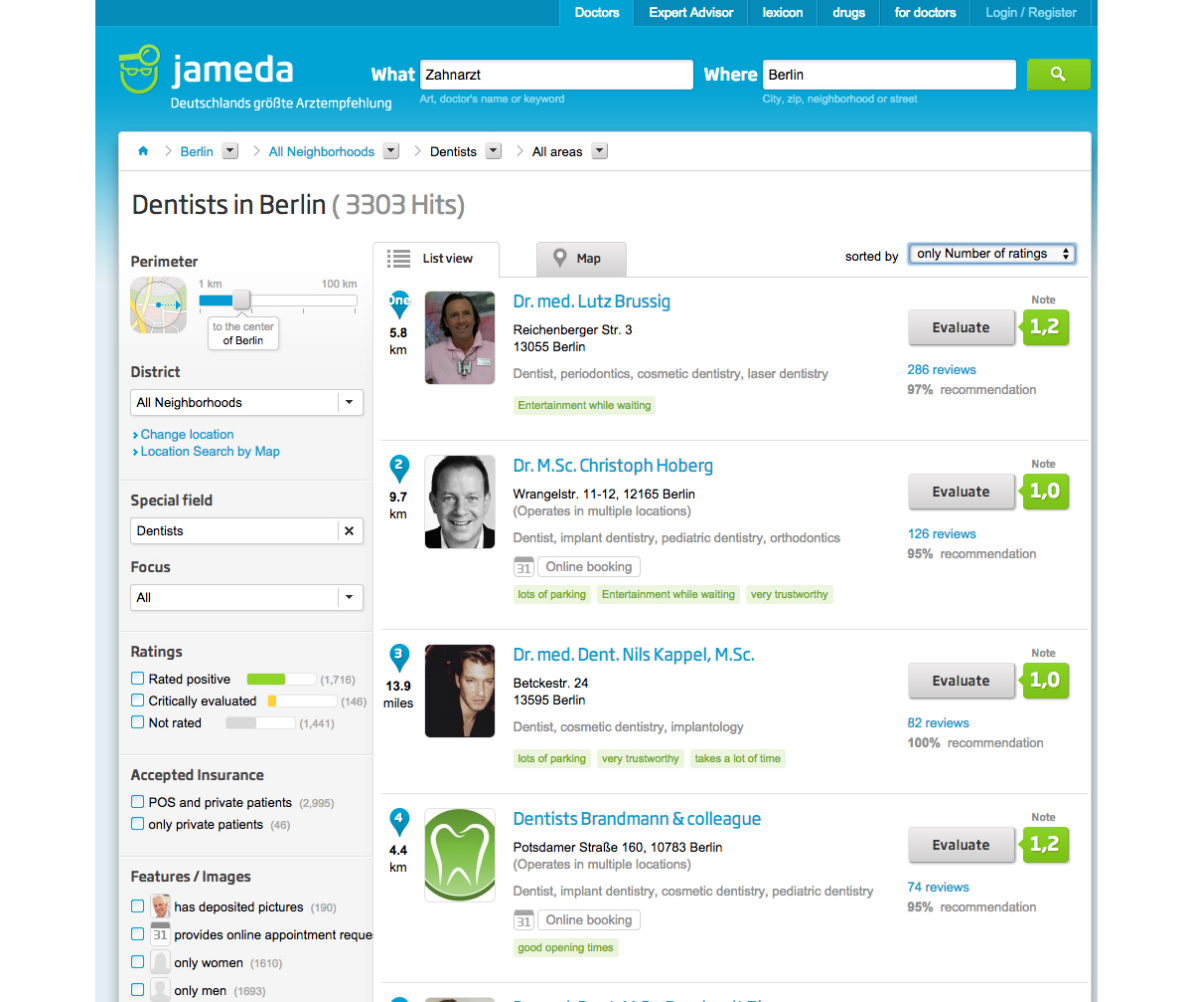
Screenshot of a dentist search on the German physician ratings website, Jameda (translated from German to English with Google Translate).

**Figure 2:**
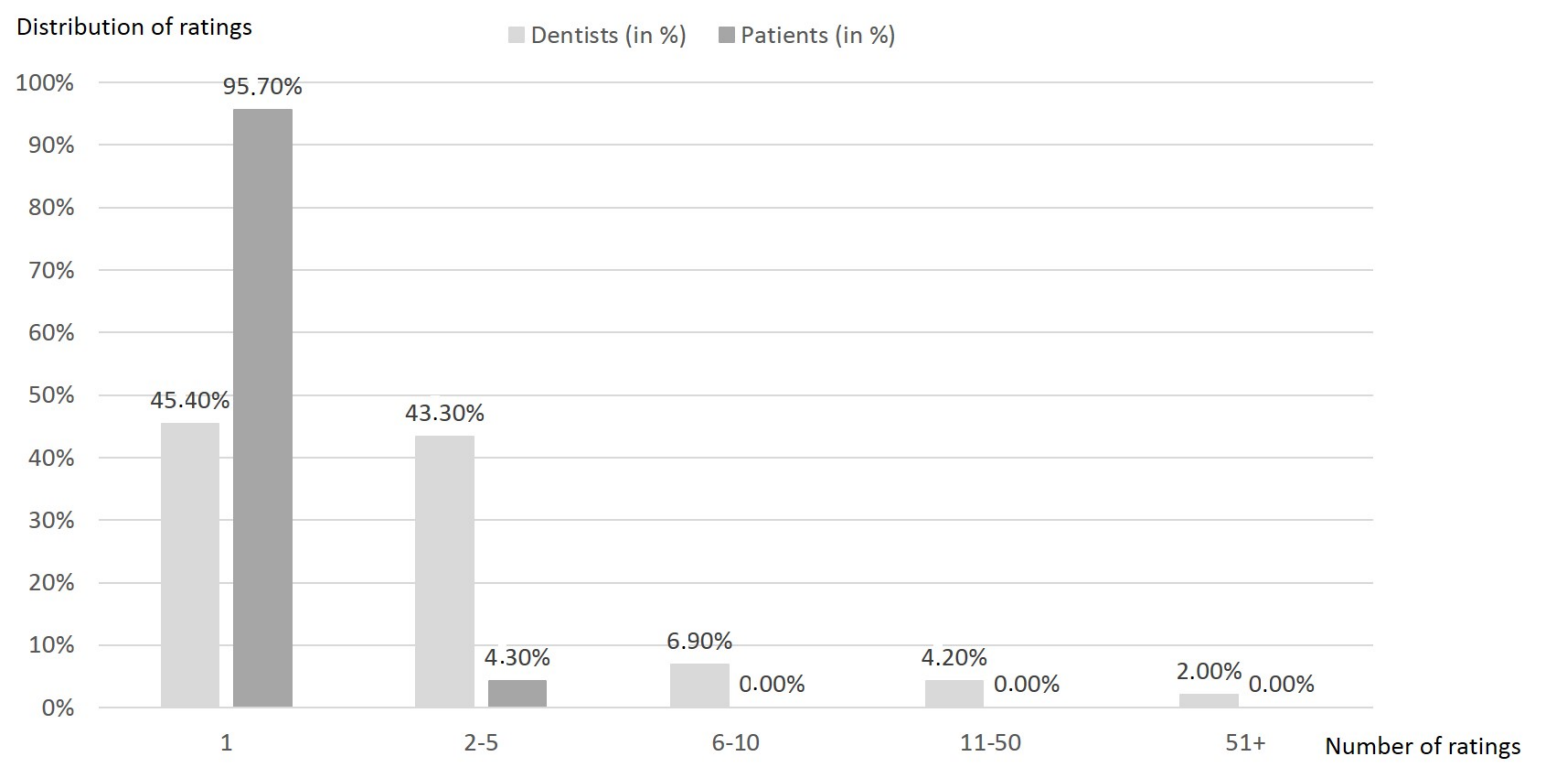
Overview of the rating distribution for both physicians and patients.

The number of dentist ratings posted on the physician rating website Jameda increased from 28,843 in 2012 to 47,613 in 2013, representing an increase of approximately 65.08%. See Figure 1 for a screenshot of a dentist search on the German physician rating website, Jameda. In 2012, the total number of dentists in Germany was 53,626 [22] and the number of rated dentists was 23,902; therefore, 44.57% (23,902/53,626) of all dentists were rated at least once within the 2-year study period. In more detail, 45.37% (10,845/23,902) of the dentists were rated once, 43.41% (10,376/23,902) received between 2 and 5 ratings, and 11.21% (2,681/23,902) were rated more than 6 times. This led to a mean 3.16 ratings per rated dentist (SD 5.57, range 1-215) (Figure 2). Most patients left only a single rating (95.67%, 69,608/72,758) and there were only a few who delivered 2 or more ratings (4.33%, 3150/72,758). The mean number of ratings was 1.05 ratings per rating patient (SD 0.25, range 1-15). Regarding the latter, this does not necessarily mean that 15 different dentists were rated; it can imply that 1 dentist was rated more than once by 1 rating patient.

The distribution of the ratings concerning dental treatment revealed that 89.21% (21,324/23,902) of the rated dentists received a 1=very good or 2=good overall evaluation, only 3.02% (721/23,902) were rated 5=deficient or 6=insufficient according to the grading system used in German schools (Table 1). The overall mean result was very good (mean 1.42, SD 0.97), ranging from 1.55 for question 3 (the relationship of trust with the dentist) to 1.37 for question 5 (the friendliness of the dentist).

**Table 1:** Evaluation results of all rated dentists on Jameda from 2012-2013 (N=23,902).

Performance range		Overall performance	Question (Q)^a^				
			Q1	Q2	Q3	Q4	Q5
**Rating, n (%)** ^b^							
	1	18,649 (78.02)	18,302 (76.57)	17,528 (73.33)	17,706 (74.08)	17,878 (74.80)	18,979 (79.40)
	2	2675 (11.19)	2846 (11.91)	3714 (15.53)	3039 (12.71)	3498 (14.63)	2848 (11.89)
	3	1262 (5.28)	1088 (4.55)	1205 (5.04)	1212 (5.07)	1181 (4.94)	974 (4.07)
	4	592 (2.48)	781 (3.27)	734 (3.07)	874 (3.66)	671 (2.81)	597 (2.50)
	5	492 (2.06)	356 (1.49)	363 (1.52)	401 (1.68)	331 (1.38)	217 (0.91)
	6	229 (1.00)	529 (2.21)	358 (1.50)	670 (2.80)	343 (1.44)	287 (1.20)
Mean (SD)		1.42 (0.97)	1.48 (1.08)	1.48 (1.01)	1.55 (1.15)	1.46 (0.98)	1.37 (0.90)
Median (range)		1.00 (1.00-6.00)	1.00 (1.00-6.00)	1.00 (1.00-6.00)	1.00 (1.00-6.00)	1.00 (1.00-6.00)	1.00 (1.00-6.00)
Skewness		2.79	2.83	2.77	2.62	2.89	3.28
Kurtosis		7.79	7.91	8.04	6.56	8.81	11.73

^a^ Q1: satisfaction with the treatment by the dentist; Q2: education about the illness and treatment; Q3: relationship of trust with the dentist; Q4: time the physician spent for the patient´s concerns; Q5: friendliness of the dentist.

^b^ German school-based rating system (1=very good; 2=good; 3=satisfactory; 4=sufficient; 5=deficient; 6=insufficient).

### Evaluation

With regard to gender, male dentists were more likely to be rated compared to their female counterparts (Table 2); 31.89% (7621/23,897) were female (the average proportion of female dentists as a percentage of all German dentists in 2012 was 37.2%). In contrast, 54.41% (26,915/49,465) of the rating patients were female and 45.59% (22,550/49,465) were male (the remainder did not provide this information). The age group of patients with the largest proportion of ratings was between 30 and 50 years (53.60%, 25,015/46,673), and most were covered by statutory health insurance (78.77%, 37,139/47,147). As shown in Table 2, female dentists received better ratings compared to their male colleagues (*P*<.001) and female patients also gave better ratings (*P*<.001). Considering the different age groups of patients, the older the patients, the higher the proportion of good ratings (*P*<.001). Furthermore, those covered by private health insurance gave more favorable ratings than those covered by statutory health insurance.

Further analysis was carried out on the basis of ratings covering all dental subdisciplines concerning the entire 2-year study period (2012 and 2013). Regarding the overall rating for dental subdisciplines, pediatric dentists and periodontologists received significantly better ratings than orthodontists did.

Table 3 illustrates a slight worsening of the ratings in 2013 in comparison with the previous year (*P*<.05). We observed a marginal decline in the ratings for female dentists in 2013 (*P*<.05). Regarding the dental subdisciplines, no significant improvement was seen. In contrast, a significant worsening for periodontologists and endodontists was determined.

**Figure 3:**
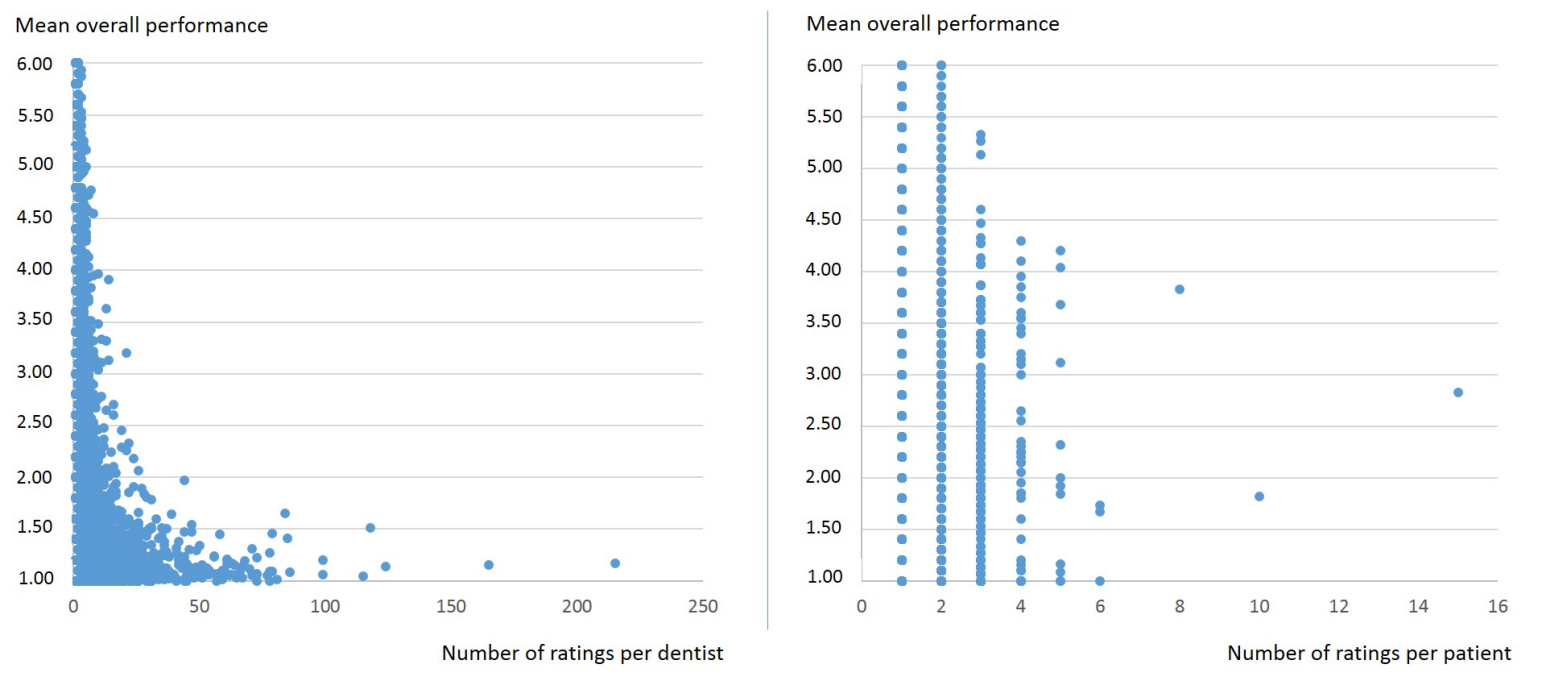
Scatterplot (bivariate) of the number of ratings per dentist (left) and patient (right) with the mean overall performance for a rated dentist.

Finally, we assessed the correlation between the mean overall performance of a dentist and the number of ratings per dentist. In Figure 3, dentists who received a higher number of ratings were shown to have statistically significant overall better ratings (Kendall tau-b=0.185, *P*<.001). This significant correlation could also be determined for all 5 mandatory questions (*P*<.001; data not presented here). A significant correlation between the number of ratings per patient compared to the mean overall performance given by this patient (Kendall tau-b=0.148, *P*<.001) was found also. Again, this was true for all 5 mandatory questions (*P*<.001; data not presented here).

**Table 2:** Rating differences for dentist and patient characteristics from 2012-2013.

Characteristics			n	>Median, n	≤Median, n (%)	*P* ^a^
**Dentist**						
	**Gender**					.001
		Female	7621	3462	4159 (54.57)	
		Male	16,276	7784	8492 (52.17)	
	**Medical specialty**					<.001
		Pediatric dentistry	84	36	48 (57.14)	
		Not specified	12,608	5535	7073 (56.10)	
		Periodontology	4556	2060	2496 (54.78)	
		Naturopathy	159	75	84 (52.83)	
		Endontology	275	132	143 (52.00)	
		Oral surgery	715	354	361 (50.49)	
		Implantology	3614	1925	1689 (46.73)	
		Esthetic dentistry	562	332	230 (40.93)	
		Orthodontics	1284	771	513 (39.95)	
		Laser dentistry	45	29	16 (35.56)	
**Patient**						
	**Gender**					<.001
		Female	26,915	6426	20,489 (76.12)	
		Male	22,550	5811	16,739 (74.23)	
	**Age**					.001
		<30	8746	2727	6024 (68.87)	
		30-50	25,009	6116	18,898 (75.56)	
		>51	12,908	2645	10,263 (79.51)	
	**Health insurance**					.001
		Statutory health insurance	37,283	9408	27,875 (74.77)	
		Private health insurance	9995	2360	7635 (76.39)	

^a^Median test: a nonparametric test that is used to determine whether the medians of two or more groups differ.

**Table 3:** Rating differences of dentists for the time period of 2012-2013.

Characteristics		2012		2013		*P*
		n	≤Median, n (%)	n	≤Median, n (%)	
**Gender**						
	Female	7711	5643 (73.18)	12,833	9221 (71.85)	.04
	Male	21,105	15,674 (74.27)	34,772	25,610 (73.65)	.11
	Total	28,843	21,342 (73.99)	47,613	34,836 (73.16)	.01
**Medical specialty**						
	Pediatric dentistry	131	113 (86.26)	298	242 (81.21)	.26
	Not specified	11,952	8738 (73.11)	18,223	13,193 (72.40)	.18
	Periodontology	4840	3561 (73.57)	7565	5437 (71.87)	.04
	Naturopathy	199	149 (74.87)	297	219 (73.74)	.86
	Endontology	464	389 (83.84)	1033	817 (79.09)	.04
	Oral surgery	1120	834 (74.46)	2051	1571 (76.60)	.19
	Implantology	6564	5018 (76.45)	11,470	8633 (75.27)	.08
	Esthetic dentistry	1662	1344 (80.87)	3352	2651 (79.09)	.15
	Orthodontics	1723	1050 (60.94)	3073	1882 (61.24)	.86
	Laser dentistry	187	145 (77.54)	250	191 (76.40)	.87

## Discussion

Currently, most patients seek advice from friends or relatives, or choose a doctor within their vicinity when searching for a physician [7,23]. This behavior, however, could be changing because of the increasing use of physician rating websites, which have been shown to be gaining in attention and importance [15]. The latter statement can be backed by the results of this study, showing an increase in the number of ratings of 65.08% within 2 years. This increase is in contrast with the attitude of German dentists toward physician rating websites. In a survey performed in 2012, only 14.4% of German dentists questioned thought that being rated on a physician rating website was of great or comparatively great relevance for their practices. However, 64.7% of the participants believed that physician rating websites will become more important in the future [24].

According to our results, 33.69% (18,069/53,626) of all German dentists have been rated at least once in 2013. Comparing this figure with the results of the previously published study [18], this percentage is only slightly lower than for other physicians in Germany (37%). Compared with additional national or international literature dealing with ratings on physician rating websites, the percentage of rated dentists rated in Germany is fairly high. According to a study by Gao and colleagues [19], approximately 16% of all doctors listed were rated on the Canadian physician rating website RateMDs between 2005 and 2010, whereas Lagu and colleagues [25] determined a percentage of 27% in the case of 300 doctors. An analysis of different German physician rating websites showed that between 3% and 28% of doctors had been rated at least once on different German physician rating websites in 2012 [21].

The mean number of ratings per dentist was 2.06 (SD 2.99) in 2012, slightly lower than for other German physicians at 2.37 [18]. Strech and Reimann [21] analyzed several German physician rating websites and calculated a mean between 1.1 and 3.9 ratings per physician. Results for websites in the United States show similar results: 2.35 [25], 2.4 [26], and 3.2 [19]. In 2012, 61.96% of dentists had been rated once; this number declined to 54.72% in 2013. Thereby, the percentage of dentists with between 2 and 5 ratings increased from 32.92% in 2012 to 36.54% in 2013. In the comparative study, 49.7% of doctors were rated only once and 43.7% of the doctors received between 2 and 5 ratings [18]. In both studies, the percentage of doctors with 10 or more ratings was less than 5%. An analysis of the United States portal, RateMDs, showed similar results; half of doctors received 1 evaluation and 12.5% were rated more than 5 times [19].

In general, these results show positive rating results for German dentists (eg, 89.21% of all ratings were in the top 2 categories). These results are in-line with the results of a nationwide survey on public attitude to, and assessment of, dental care in Germany. This indicates that dentists enjoy a very good reputation. On a 4-stage scale, 91% of the people questioned are either “rather satisfied” or “very satisfied” with their dentist [4]. In another study addressing patient perspective on the quality of oral health care in Germany, the overall satisfaction with dental care was 4.66 (SD 0.55) on a 5-point scale ranging from 1 (poor) to 5 (excellent) [27]. Other studies have also previously shown a tendency toward good ratings on physician rating websites in Germany [18] and in the United States [19,25,26]. The results presented by MacKay and colleagues [28] were slightly lower; 70% of the comments on RateMDs were favorable toward Canadian physicians. Similar results were provided from a study on the usage of NHS Choices, a United Kingdom government website that encourages patients to rate the quality of physician practices. Between October 2009 and December 2010, approximately 61% of family practices were rated and the practice was recommended in 69% of the ratings [11]. According to the authors, governmental websites may create a selection bias toward less satisfied patients.

In our study, the median score of all questions was 1.00 (mean 1.42) which is in-line with the other literature. Strech and Reimann [21] calculated an average score of between 1.1 and 1.5 on a 3-stage scale (1=good; 3=bad) for different German physician rating websites. In the comprehensive analysis conducted by Kadry and colleagues [3] regarding the 10 most frequently visited physician rating websites in the United States, a mean rating of 77 (based on a 100-point scale), 3.84 (based on a 5-point scale), and 3.1 (based on a 4-point scale) was shown.

Female dentists received better ratings. This might be explained by the fact that female physicians engage in significantly more active partnership behaviors, positive talk, psychosocial counseling, psychosocial question asking, and emotionally focused talk. Medical visits were shown to be 2 minutes longer with female physicians than those with male physicians [29]. Furthermore, female patients tend to evaluate more positively compared to their male counterparts. Götz and colleagues [27] also found that female patients showed a higher overall satisfaction with dental care than male patients did. This is in contrast to another German analysis of physician rating websites showing that male patients tended to rate more positively [30]. Furthermore, it could be shown that privately insured patients tend to give better ratings more often than those covered by statutory health insurance do (*P*<.001). One reason for this could be seen in the easier access to medical services. In-line with the comparative study, middle-aged patients (aged between 30-50 years) gave the most ratings [18]. Furthermore, better ratings were most likely to be seen in the oldest age group (older than 50 years), which is similar to findings in another study showing higher overall satisfaction scores with oral health care in older patients [27].

Furthermore, our analysis revealed a slight but significant deterioration in ratings (*P*<.05). Regarding the dental subdisciplines, only periodontologists and endodontists showed a slight but significant decline in rating level. The reason for this change cannot be derived from the data available. In 2012 and 2013, orthodontists (who generally treat children and youths) were rated lowest. One possible explanation might be that orthodontic treatment is a long-term process that is often partly or fully financed privately. The copayment might lead to dissatisfaction and consequently lower ratings. In their survey with more than 8000 patients about the quality of oral health care, Götz and colleagues [27] found the domain “costs of care” was rated least positively and they subsequently recommended adequate explanation and information about treatment costs as an essential aspect of dental care. However, noncompliance by patients leading to unsatisfactory results should also be considered a possible reason. According to the estimations of two-thirds of German orthodontists, 30%-60% of their patients develop problems of cooperation during the orthodontic treatment. Up to 15% of patients even cancel their therapy [31]. Another explanation for the relatively low ratings for orthodontists might be found in the rating patients’ expectations. It might add value in this context to assess whether the ratings were given by the children themselves or their parents. In case the parents left a rating, one might argue that parents want the very best for their children and could be disappointed much more easily compared to when they visit a doctor for themselves. In contrast, children might be more critical because they want to have good-looking teeth (eg, no braces), preferably in a very short period of treatment. This, however, is not feasible because orthodontic corrections need time. Regarding the age distribution of the rating patients, the latter seems to be more likely. The proportion of rating patients aged 30 years or younger is by far the highest among all dental subdisciplines. Here, approximately 44.74% of the rating patients were aged 30 years or younger, whereas only 6.95% were aged 50 years or older (see Table 4).

**Table 4:** Age distribution of the rating patients according to the dental subdisciplines (N=20,194).

Dental discipline	Age range (years), n (%)		
	<30	30-50	≥50
Pediatric dentistry	13 (16.46)	51 (64.56)	15 (18.99)
Not specified	1531 (18.24)	4634 (55.21)	2228 (26.55)
Periodontology	574 (16.70)	1948 (56.68)	915 (26.62)
Naturopathy	24 (15.29)	77 (49.04)	56 (35.67)
Endontology	64 (18.88)	202 (59.59)	73 (21.53)
Oral surgery	169 (20.86)	397 (49.01)	244 (30.12)
Implantology	733 (15.78)	2489 (53.57)	1424 (30.65)
Esthetic dentistry	192 (17.07)	664 (59.02)	269 (23.91)
Orthodontics	476 (44.74)	514 (48.31)	74 (6.95)
Laser dentistry	24 (16.78)	89 (62.24)	30 (20.98)
Total	3801 (18.82)	11,065 (54.79)	5328 (26.38)

The limitations to this study are as follows. Although the study was based on a large dataset and its results are in-line with other studies, the major limitation is the fact that only 1 physician rating website was included in our analysis; evaluations of other physician rating websites could lead to different results. Future studies should aim at comparing different German physician rating websites and analyzing the current number of ratings and the number of physicians rated, but also differences in rating patients and rated physicians. Applying those criteria might lead to other “most important” German physician rating websites. Unfortunately, a more in-depth analysis regarding the use of physician rating websites according to sociodemographic data was not possible because of a lack of data. In this context, a recently published study analyzing the variables which influence the usage of physician rating websites could demonstrate that sociodemographic variables alone do not sufficiently predict use or nonuse of physician rating websites; specific psychographic variables and health status additionally need to be taken into account [32]. Because rating is anonymous, rating values are not risk-adjusted and, therefore, vulnerable to fraud. People providing feedback on health care services via social media are presumably not always representative of the patient population in general. Next, we were not able to present an analysis over a longer period of time. However, our study is the first evidence from Germany covering 2 consecutive years. Finally, the purpose of this paper was to give further insight into the nature of dentists’ ratings, not to discuss the usefulness of the ratings for the patients when seeking a dentist. In this context, several studies have raised concerns about the helpfulness and support of such sites from a patient’s perspective [33-35]. A comprehensive overview of the major shortcomings of physician rating websites (eg, incomplete databases, low percentage of rated physicians, the potential to reflect the quality of care) was also recently provided by a systematic review [36].

In conclusion, an increasing trend toward the rating of physicians on a physician rating website in Germany is shown. The total number of ratings rose by 65.08% between 2012 and 2013. So far, 44.57% of all German dentists have been evaluated by patients at least once. However, this means that more than half of dentists in Germany are still without any evaluation. The increase in ratings over the 2-year study period indicates a growing interest among the population to evaluate the quality of oral health care providers. Many of the results presented here are in-line with the other national and international literature in this area. The overall satisfaction of patients with dentists seems to be comparable to other medical specialties. In a patient-centered approach, it is essential to assess the quality of health care from a patient’s perspective. Information available on social media (such as physician rating websites) has gained more attention in the literature recently (eg, [9,19,20]). Therefore, they may possibly have a positive effect on the encouragement of health competence and equal opportunities of patients [21]. So far, it remains unclear whether and how online rating may reflect the technical quality of care, the measuring of patient experiences and satisfaction, and how care is being assessed. Nevertheless, the existence of physician rating websites is likely to continue and will remain an important aspect of oral health care evaluation. physician rating websites might contribute to reducing the lack of publicly available information on the quality of care [18]. Future research should explore whether social media, such as physician rating websites, are suitable in practice for patients, health insurers, and governments to help them evaluate the quality of performance of medical professionals [16].

## Abbreviations

NHS: National Health Service
